# Amino acid profile changes during enrichment of spheroid cells with cancer stem cell properties in MCF‐7 and MDA‐MB‐231 cell lines

**DOI:** 10.1002/cnr2.1809

**Published:** 2023-04-24

**Authors:** Zahra Ghanbari Movahed, Maryam M. Matin, Kamran Mansouri, Sajjad Sisakhtnezhad

**Affiliations:** ^1^ Department of Biology, Faculty of Science Ferdowsi University of Mashhad Mashhad Iran; ^2^ Novel Diagnostics and Therapeutics Research Group, Institute of Biotechnology Ferdowsi University of Mashhad Mashhad Iran; ^3^ Medical Biology Research Center Kermanshah University of Medical Sciences Kermanshah Iran; ^4^ Department of Biology, Faculty of Science Razi University Kermanshah Iran

**Keywords:** amino acid metabolism, breast cancer, cancer stem cells, MCF‐7, MDA‐MB‐231, spheroid culture

## Abstract

**Background:**

Cancer stem cells (CSCs), subpopulations of cancer cells, are responsible for tumor progression, metastasis, and relapse. Changes in amino acid metabolism are linked to breast cancer recurrence and metastasis.

**Aims:**

This study aimed to evaluate the changes in the amino acid profile in MCF‐7 and MDA‐MB‐231 cells during spheroid formation to discover the specific metabolic properties in CSCs.

**Methods:**

MCF‐7 and MDA‐MB‐231 breast cancer cells were cultured as spheroids and evaluated to characterize their CSC properties. The characteristics of CSC were evaluated by examining the expression of CSC markers and conducting drug resistance assays. In addition, amino acid profile change during the enrichment of breast cancer stem cells in the spheroids was investigated by high‐performance liquid chromatography (HPLC).

**Results:**

The results indicated that out of 20 different amino acids analyzed, 19 of them decreased during the spheroid formation process. Alanine, lysine, phenylalanine, threonine, and glycine showed significant reductions in the conditioned media of both cell lines in the spheroid form compared to the monolayer cells. Only one of the amino acids increased in MCF‐7 and MDA‐MB‐231 spheroids (histidine and serine, respectively).

**Conclusion:**

Our results suggest that certain amino acids identified in this study can be used for a better understanding of the molecular mechanisms associated with breast cancer stem cell formation.

## Graphical abstract

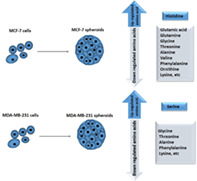

## 1 INTRODUCTION

Breast cancer is the second most regularly diagnosed cancer in women, with 1.38 million cases (10.9%) being diagnosed globally.^1^ Unfortunately, as a result of applying standard therapies with a moderate success rate, most patients experience relapses with hormonal‐resistant breast cancer, which can eventually advance to metastasis.^2^ A small subpopulation of cells, known as Cancer Stem Cells (CSCs), resides in a tumor. These cells are responsible for the metastasis and tumorigenesis properties of the cancer cells.^3^, ^4^, ^5^, ^6^ CSCs are categorized by their slow cell cycle, resistance to chemotherapy and oxidative stress, and rapid response to genotoxic damages.^7^, ^8^, ^9^, ^10^

Many studies have tried interfering with various metabolic pathways in tumors to eradicate cancer cells.^11^, ^12^, ^13^, ^14^ Compared to other metabolic pathways, the amino acid metabolic network is more complicated and deeply connected with other pathways.^15^ Specifically, amino acid metabolism has a significant role in cancer cell behavior under oxidative, nutritional, and genotoxic stresses.^16^ Thus, we can consider targeting amino acid metabolism as a potential therapeutic strategy for cancer patients.

Metabolic reprogramming in CSCs has a significant role in these cells adapting to changes in the tumor microenvironment and maintaining their unlimited self‐renewal ability.^17^, ^18^

Therefore, we developed spheroids from MCF‐7 and MDA‐MB‐231 breast cancer cell lines to investigate their amino acid contents after comprehensively characterizing their CSC properties. We used HPLC to compare the amino acid profiles of these CSC‐enriched spheroids in contrast to the parental cells.

## 2 MATERIALS AND METHODS

### 2.1 Cell culture

MCF‐7 and MDA‐MB‐231 breast cancer cell lines were procured from the Pasteur Institute, Iran. All cell lines were tested for mycoplasma contamination and confirmed using STR profiling at the Pasteur Institute of Iran. The cells were cultivated in Dulbecco's modified Eagle's medium/nutrient mixture F‐12 (DMEM/F12) (Cat. No. 12500096, Gibco, UK) complemented with 10% fetal bovine serum (FBS) (Cat. No. A5256701, Gibco, UK) and 1% penicillin/streptomycin (P/S) (Cat. No. 15070‐063, Gibco, UK). Cells were grown at 37°C in a humidified atmosphere of 95% air/5% CO_2_. Upon 80% confluency, cells were subcultured in a fresh medium using trypsin–EDTA.

### 2.2 Sphere formation assay (SFA)

To assess the anchorage‐independent colony formation capacity of CSCs, a soft agar assay was performed. In brief, 1% (g/mL) agarose powder (Cat. No. 16550100, Invitrogen) was mixed with distilled water and autoclaved for sterilization. Then, we added 2 mL of the agarose solution to the wells of 6‐well plates (Cat. No. 220100, Sorfa, Malaysia). After solidification, single‐cell suspensions of MCF‐7 and MDA‐MB‐231 cells were thoroughly suspended and placed in agarose‐coated 6‐well plates at 5 × 10^4^ cells/well density in 2 mL of sphere formation media. The culture dishes were stored at 37°C in a CO_2_ incubator. The culture medium contained DMEM/F12, 5% HS (human serum), and 1% P/S. Colonies were formed after 2 days of growth. Following 48 h of culture, the primary sphere formation was assayed under an inverted microscope (OPTIKA SN263975; Italy). Spheres were defined as cell colonies >50 μm in diameter, showing a 3‐dimensional structure and beclouded cell margins.

### 2.3 Morphology assessment

Spheroid formation was recorded using an inverted microscope. The cell morphological changes subjected to drug sensitivity assays were also subjectively explored via microscopic evaluation.

### 2.4 Drug sensitivity assay

The MTT (3‐(4,5‐dimethyl‐2‐thiazolyl)‐2,5‐diphenyl‐2*H*‐tetrazolium bromide) assay (Cat. No. 11465007, Sigma‐Aldrich, USA) was applied to assess the sensitivity of spheroids and monolayer cells to a chemotherapeutic drug. The monolayer and spheroid cells were seeded in 96‐well microplates using a similar cell culture medium. However, for spheroids, the single cells were placed in a 96‐well plate coated with agarose. For the monolayer and spheroid cultures, cells were detached into individual cells, counted, and seeded at 5 × 10^3^ cells/well density in a 96‐well plate. Cultures were then treated with 200 μL/well of doxorubicin (EBEWE Pharma, Austria) at different concentrations (0–100 μg/mL). The diluent (DMEM) alone was used as the control. Standard MTT assay for monolayer cells^19^ and a slightly modified version for spheroids,^20^ were applied. After 24 h of treatment, 20 μL of MTT solution (5 mg/mL in PBS) was added to the spheroid or monolayer cell cultures, followed by incubation for 3 h. The monolayer culture in the original plate was left intact, but the well content composing of the spheroid culture was transferred into a new, flat‐bottom plate of 96 wells before the plate centrifugation at 600 *g* for 5 min. The medium was then removed from each well of both plates composed of the spheroid or monolayer cultures. Then, the plates were spot‐dried on a napkin, and 200 μL of DMSO was added to dissolve the formazan crystals. Afterward, the absorbance of each well was read in a microplate reader (Awareness Technology, USA) at 570 nm while subtracting the absorbance of the background at 630 nm. The cytotoxicity was determined by the mean absorbance of the treated cells compared to controls (regarded as 100% viability) and considered as cell viability percentage. The appearance of the treated spheroids was visualized using an inverted microscope at different drug concentrations 24 h post‐treatment. Untreated cells were cultured parallel to the experiments, which were all carried out in triplicate.

### 2.5 RNA extraction and real‐time PCR analysis

After 48 h of incubating MCF‐7 and MDA‐MB‐231 cells in monolayer and spheroid forms at 37°C in a humidified atmosphere of 95% air/5% CO_2_, RNAX‐Plus (Cinaclon, Iran) was used to extract the total RNA content from cells according to the manufacturer's instructions. Reverse transcription‐PCR was performed using Exce lRT™ Reverse Transcription Kit (Cat. No. RP1400, SMOBIO, Taiwan). Then, the real‐time PCR reactions were carried out using a Rotor‐Gene 6000 system (Corbett Research, Australia), and the YTA SYBR Green qPCR Master Mix (Cat. No. YT2551, Yekta Tajhiz Azma, Iran). Then, mRNA expression was quantified using the delta CT method, and beta‐actin served as the internal control. The following primers were used:

*NANOG*: forward 5′‐TGAGATGCCTCACACGGAGAC‐3′ and reverse 5′‐GGTTGTTTGCCTTTGGGACTG‐3′; *OCT*‐*4*: forward 5′‐GGTGCCTGCCCTTCTAGGAATG‐3′ and reverse 5′‐TGCCCCCACCCTTTGTGTTC‐3′; *Beta*‐*ACTIN*: forward 5′‐ACCTTCTACAATGAGCTGCG‐3′ and reverse 5′‐ CCTGGATAGCAACGTACATGG‐3′.

### 2.6 Flow cytometric analysis

Flow cytometry was performed to investigate CD44 and CD24 expression in MCF‐7 and MDA‐MB‐231 cells. For CD44 and CD24 expression analysis, 1 × 10^6^ cells were suspended in 100 μL PBS/2% BSA (bovine serum albumin) and incubated with fluorescence isothiocyanate (FITC)‐conjugated antibody against CD44 (1:20) (Cat. No. 555749, BD Pharmingen, USA) and phycoerythrin (PE)‐conjugated antibody against CD24 (1:20) (Cat. No. 561647, BD Pharmingen, USA) in the dark at 4°C for 30 min. Then, after washing the cells with PBS, they underwent centrifugation for 5 min at 800 *g*. Individual‐cell suspensions were evaluated using an Attune NxT flow cytometer (Thermo Fisher Scientific, USA) and FlowJo software (Version 10; Tree Star, Inc., Ashland, OR).

### 2.7 Preparation of conditioned media

Both cell lines were suspended in DMEM‐F12 containing 5% HS (human serum) and 1% penicillin/streptomycin. Regarding monolayer culture, 500 μL of the cell suspensions were seeded in a 24‐well plate at 3 × 10^5^ cells/well density for 48 h. For spheroids, 500 μL of the cell suspensions (3 × 10^5^ cells/well) were seeded in a 24‐well plate coated with agarose for 48 h. After 48 h, spheroid and monolayer cells were washed with PBS and then the culture medium of cells was replaced with 500 μL of the serum‐free medium. After 24 h, 200 μL of the conditioned medium was collected from both monolayer and spheroid cultures and centrifuged at 200 *g* for 10 min, followed by storing at −80°C. The control group for the experiment was 200 μL of the culture medium (DMEM‐F12) without serum, which underwent incubation in the incubator for 24 h and then stored at −80°C.

### 2.8 Changes in amino acid profiles

For each 1 mL of supernatant, 0.1 mL trichloro acetic acid (TCA) (Cat. No. 76‐03‐9, Sigma Aldrich, USA) was added to precipitate proteins. Then, centrifugation was carried for 10 min at 600 *g*. The supernatants were collected and placed on ice for HPLC analysis of amino acids.

Substance quantification in the cell‐free microenvironment was done using HPLC analysis with the YL9100 system (Young Lin, Korea). Chromatographic separation of amino acids was performed using a C18 column (25 cm long × 4.0 mm i.d., 5 μm, GL Sciences, Japan).

### 2.9 Statistical analysis

Values are reported as the mean ± standard deviation at 95% confidence intervals of a minimum of three separate experiments. Data analysis was performed by independent sample *t*‐test and two‐way analysis of variance (ANOVA) using GraphPad Prism 8.0.2. *p*‐value <.05, *p*‐value <.01 and *p*‐value <.001 was considered as significant.

## 3 RESULTS

### 3.1 The formation of MCF‐7 and MDA‐MB‐231 spheroids

**FIGURE 1:**
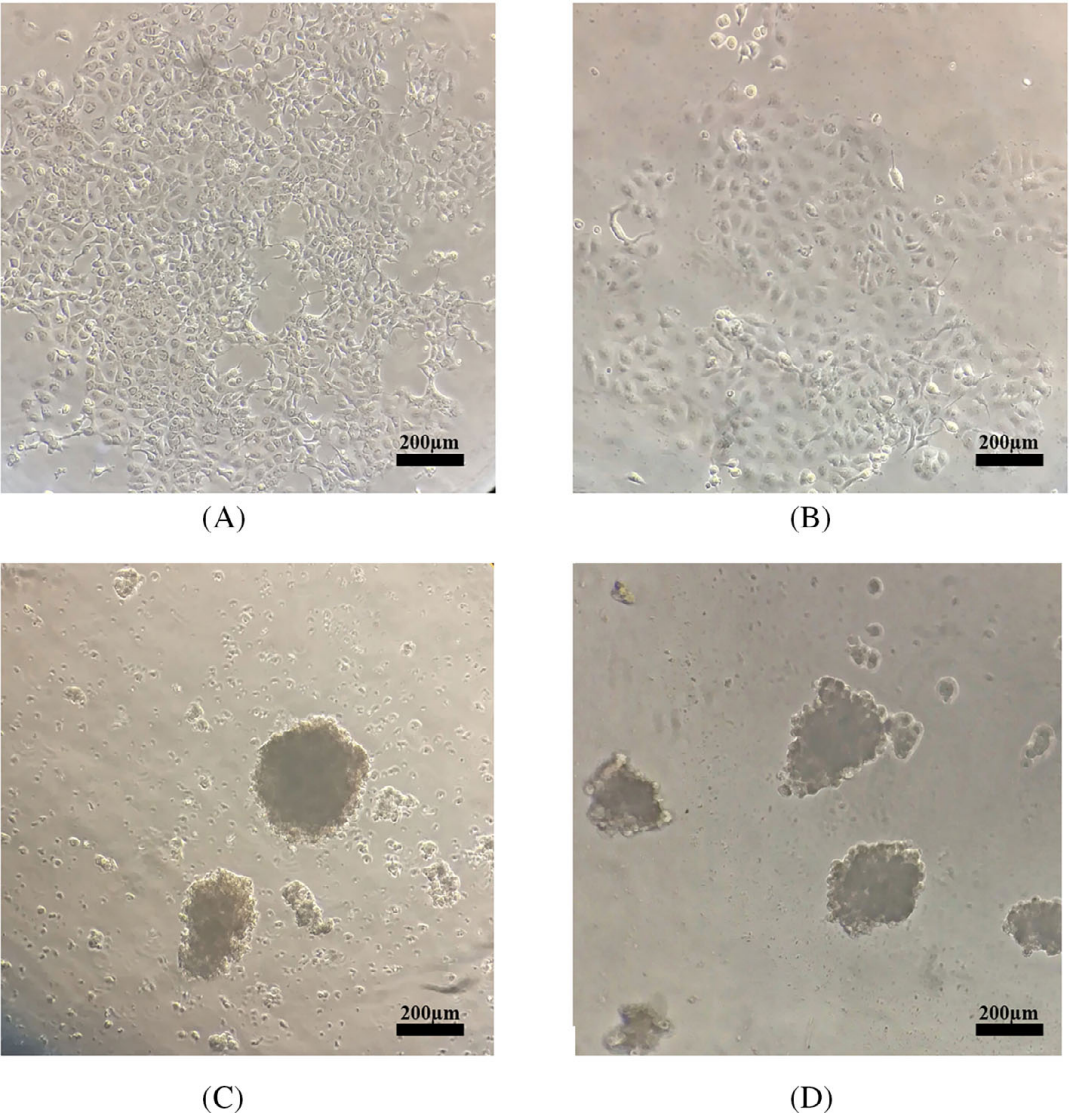
The morphology of MCF‐7 and MDA‐MB‐231 monolayer and spheroid cultures. Morphology of (A) MCF‐7 and (B) MDA‐MB‐231 monolayers under the 2D condition in tissue culture flasks using an inverted microscope (magnification: 25×, scale bar: 200 μm). The cells were found to be adherent and epithelial. The appearance of (C) MCF‐7 and (D) MDA‐MB‐231 spheroidal structures under the inverted microscope 48 h post‐culture (magnification: 25×, scale bar: 200 μm).

During monitoring under an inverted microscope in normal conditions, we detected MCF‐7 and MDA‐MB‐231 cells as monolayers of adherent epithelial‐like cells with polygonal appearance and sharp and clear boundaries (Figure 1A,B). In the spheroid induction condition and compared to their monolayer counterparts, both cell lines formed spheroids after 24–48 h. The agar‐coated microplate wells induced the generation of single spheroids of reproducible size. The spheroids formed after 48 h of culture (Figure 1C,D).

### 3.2 MCF‐7 and MDA‐MB‐231 spheroids exhibited higher drug resistance against conventional chemotherapy

**FIGURE 2:**
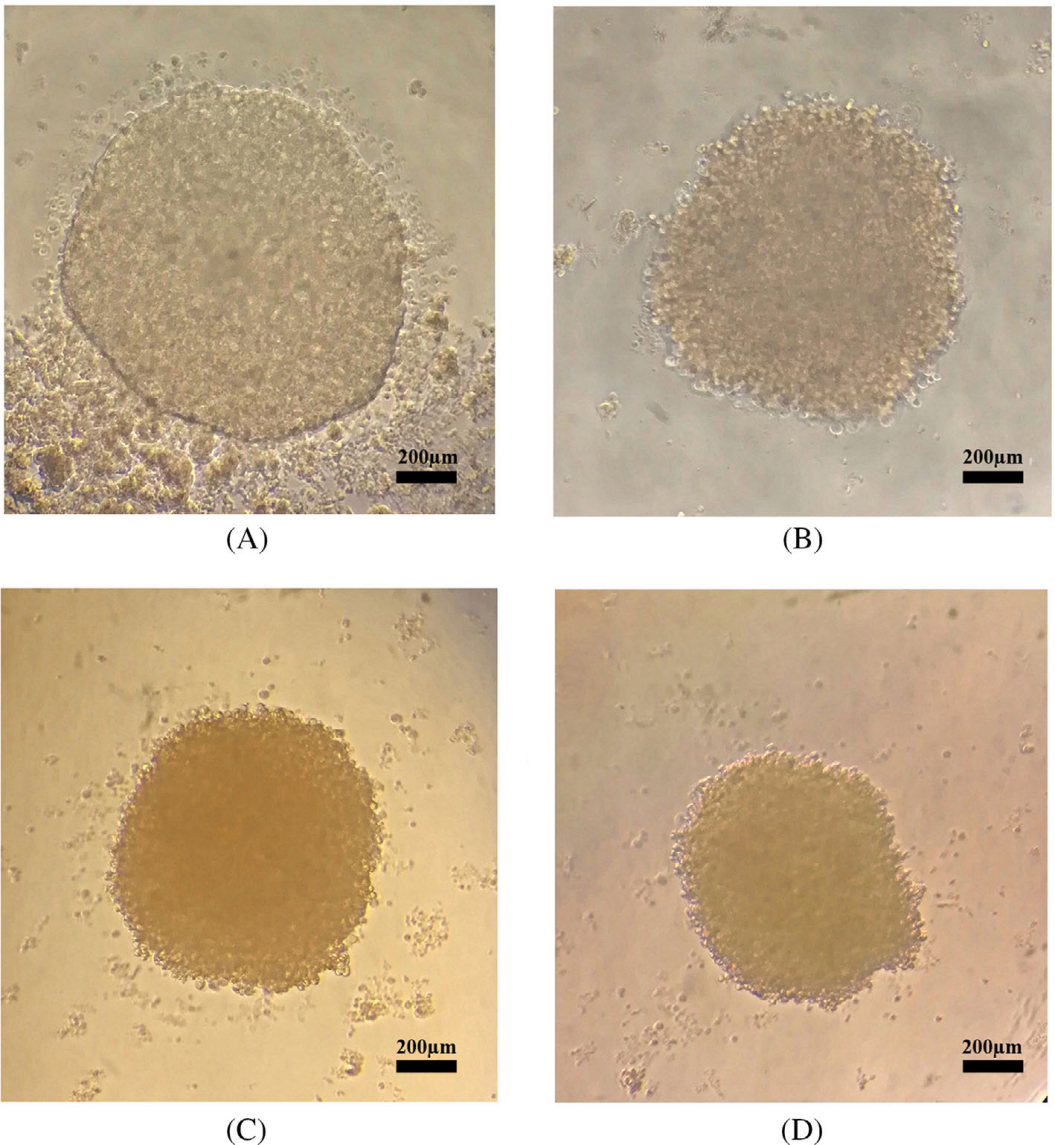
Spheroid integrity after treatment with doxorubicin chemotherapeutic drug at 30 μg/mL concentration. Compared to (A) MCF‐7 and (B) MDA‐MB‐231 parental cells, (C) MCF‐7 and (D) MDA‐MB‐231 spheroids showed higher drug resistance against conventional chemotherapy. The spheroids and parental cells received doxorubicin for 24 h. Each experiment was done in triplicate, and only the represented images are displayed. Magnification at 25×, scale bar: 200 μm.

We evaluated anticancer drug sensitivity using monolayer and spheroid cells cultured in different concentrations of doxorubicin. We cultured MCF‐7 and MDA‐MB‐231 cell lines in 96‐well plates (in monolayer and spheroid forms) and assayed them after drug treatment. Overall spheroid cultures were more resistant to drug treatment than monolayer cells (Table 1). Microscopic imaging that we carried out on spheroids 24 h after treatment at different drug concentrations showed more rigid and compact spheroids (Figure 2), indicating the possibility of CSC enrichment in the spheroid cultures is associated with higher drug resistance compared to monolayer cells.

**TABLE 1:** The IC_50_ values (μg/mL) of doxorubicin on MCF‐7 and MDA‐MB‐231 parental and spheroid cells 24 h post‐treatment.

	IC50	
	MCF‐7	MDA‐MB‐231
Spheroid cells	92.84 ± 1.27*	81.65 ± 5.32*
Monolayer cells	24.75 ± 3.25*	12 ± 7.21*

*Note*: Cell survival was determined by MTT assay. All data are expressed as means ± SD.

* *p* < .05 compared to the parental cell.

### 3.3 Enrichment of CD44 ^+^/CD24 ^−^ cells in spheroids of MCF‐7 and MDA‐MB‐231 cells

**FIGURE 3:**
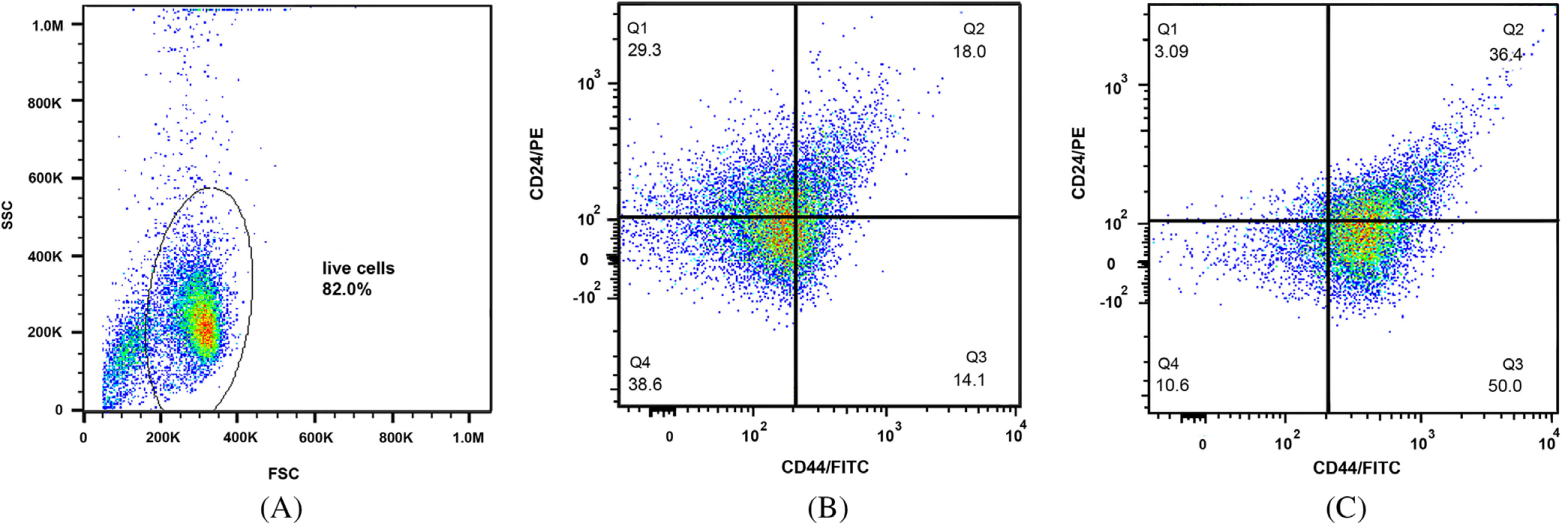
Flow cytometry analysis of MCF‐7 spheroids compared to their parental cells. (A) The percentage of the live cells, (B) the percentages of the CD44^+^/CD24^−^ subpopulations of MCF‐7 parental, and (C) spheroid cells. The proportion of CD44^+^/CD24^−^ cells in MCF‐7 spheroids was significantly higher than the parental cells.

**FIGURE 4:**
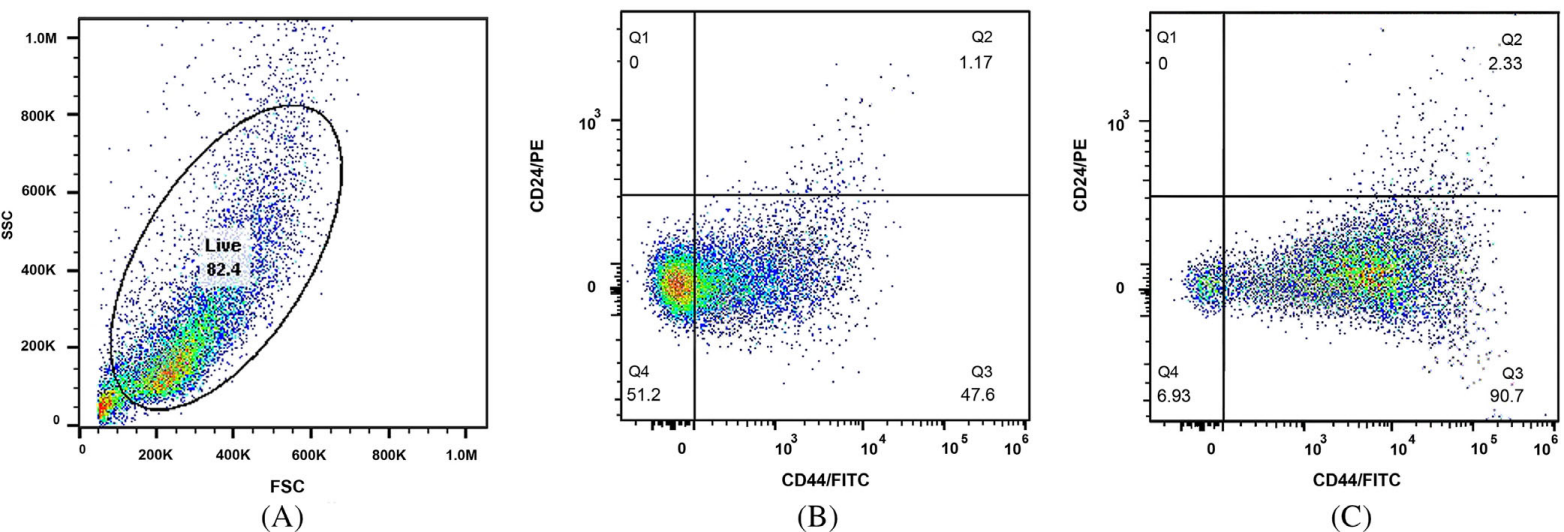
Flow cytometry analysis of MDA‐MB‐231 spheroids compared to their parental cells. (A) The percentage of the live cells, (B) the percentages of the CD44^+^/CD24^−^ subpopulations of MDAMB‐231 parental, and (C) spheroid cells. The proportion of CD44^+^/CD24^−^ cells in MDA‐MB‐231 spheroids was significantly higher than the parental cells.

CD44, as a cell surface glycoprotein, is associated with cell migration, cell adhesion, and cell–cell interactions. In addition, CD44 is considered a cell surface marker for several prostate and breast CSCs.^21^ Additionally, CD24 is involved in many cancer metastases, and its expression is associated with differentiated epithelial characteristics.^22^ Therefore, we analyzed the spheroid cultures of MCF‐7 and MDA‐MB‐231 for their CSC properties based on the expression of such stem cell‐related markers for breast CSCs. We assessed CD44 and CD24 subpopulations in spheroids and monolayer cells with a flow cytometer. The CD44^+^/CD24^−^ MCF‐7 cells in spheroids and the parental counterparts constituted 50.0% and 14.1% of cells, respectively (Figure 3). The CD44^+^/CD24^−^ MDA‐MB‐231 cells in spheroids and monolayer form accounted for 90.7% and 47.6% of cells, respectively (Figure 4). These results suggested that in comparison with monolayer cultures of both cell lines, spheroids showed a significant enrichment with CD44^+^/CD24^−^ cells under spheroid‐induced conditions.

### 3.4 Spheroids expressed cancer stem cell markers

**FIGURE 5:**
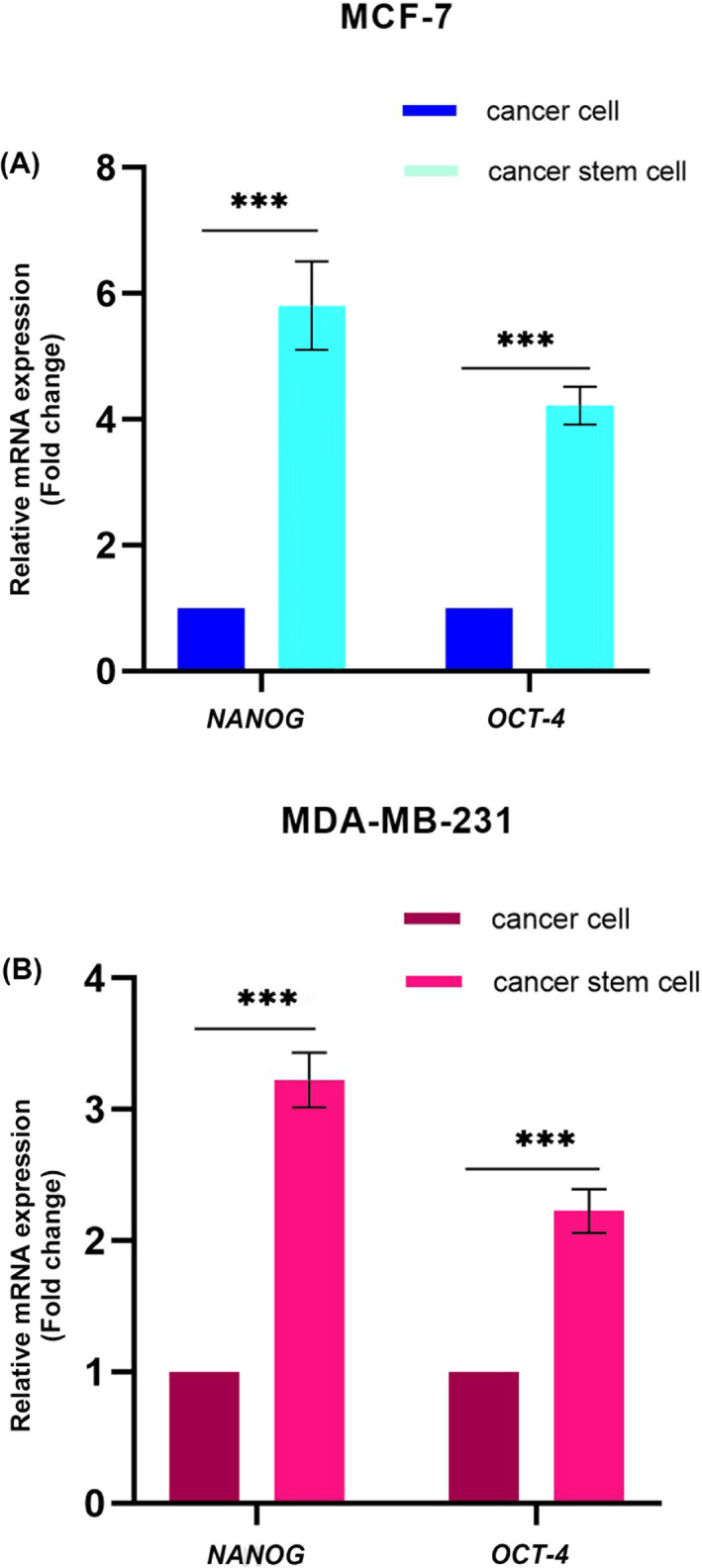
Transcript levels of *NANOG* and *OCT*‐*4* in (A) MCF‐7 and (B) MDA‐MB‐231 spheroids compared to monolayer cells as quantified by real‐time PCR, ***p < .001.

As an additional confirmation of the MCF‐7 and MDA‐MB‐231 spheroids' stemness, we measured the relative gene expression of stem cell markers by real‐time PCR. The relative gene expression of two stem cell markers was significantly higher (*NANOG*: 5.8 folds and *OCT‐4*: 4.2 folds in MCF‐7, and *NANOG*: 3.2 folds and *OCT‐4*: 2.2 folds in MDA‐MB‐231) spheroids compared to the adherent cells (Figure 5A,B).

Overall, all the tested stem cell markers confirm that MCF‐7 and MDA‐MB‐231 cells under spheroid culture condition enabled the enrichment of stem‐like cells compared to the standard adherent cell culture.

### 3.5 Amino acid profile change during spheroid formation

**FIGURE 6:**
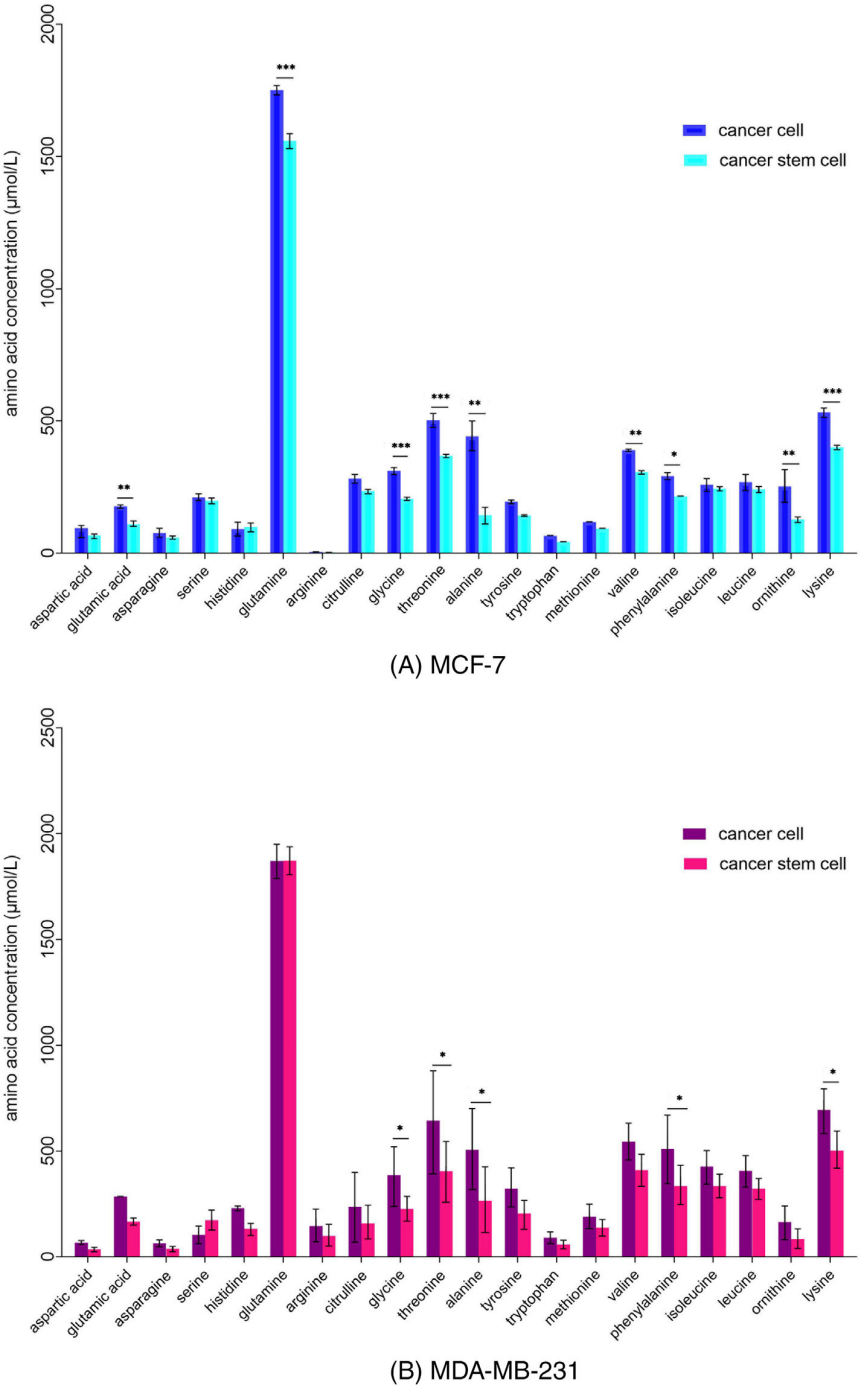
Comparing amino acid profiles between cancer cells and CSCs in (A) MCF‐7 and (B) MDA‐MB‐231. *p < .05; **p < .01; ***p < .001.

Amino acid profiles altered in MCF‐7 and MDA‐MB‐231 spheroid‐conditioned media, compared to the monolayer‐conditioned media (Figure 6). Alterations were observed as a decrease or an increase in amino acids in conditioned media. Out of the 20 different amino acids analyzed, 19 amino acids decreased during the spheroid formation process in both cell lines. The results demonstrated a significant decrease of glutamic acid, glutamine, glycine, threonine, alanine, valine, phenylalanine, ornithine, and lysine in the MCF‐7 spheroid‐conditioned media. Media conditioned with MDA‐MB‐231 spheroid cells indicated a significant decrease of glycine, threonine, alanine, phenylalanine, and lysine and a non significant increase in serine. In addition, only one amino acid increased in MCF‐7 and MDA‐MB‐231 spheroids (histidine and serine, respectively), however, they were not significant.

## 4 DISCUSSION

Although amino acid metabolism pathways have a crucial effect on the CSC properties,^23^ very few studies have assessed the amino acid profile of CSCs in different cancers. In this study, we investigated the amino acid profile of the conditioned media of MCF‐7 and MDA‐MB‐231cells in spheroids with CSC properties and monolayer cultures. The variation in these profiles could render a potent groundwork for further cancer research for understanding mechanisms involved in the formation of breast cancer stem cells.

In the present study, 20 amino acids in the conditioned media were assessed during CSCs‐enrichment in MCF‐7 and MDA‐MB‐231 cell lines. Studies have shown that these amino acids are associated with CSC properties, such as survival, tumorigenesis, self‐renewal, stemness, metastasis, and chemoresistance.^23^

Studies have shown that amino acid uptake increases in the CSC population.^16^, ^24^, ^25^, ^26^, ^27^, ^28^, ^29^, ^30^ In our study, out of the 20 different amino acids analyzed, 19 amino acids decreased during the spheroid formation process in MCF‐7 and MDA‐MB‐231 conditioned media, indicating an increase in the uptake of these amino acids.

Our results also showed that the amount of serine in the culture medium of MDA‐MB‐231 spheroids was higher than the monolayer cells. The changed amino acid profiles in MCF‐7 and MDA‐MB‐231 media potentially reflected different characteristics in these two cell lines. MCF‐7 is an estrogen receptor (ER)‐and a progesterone receptor (PR)‐positive cell line, which belongs to the luminal A molecular subtype. MDA‐MB‐231 is a highly aggressive, invasive, and poorly differentiated triple‐negative breast cancer (TNBC) cell line as it lacks ER and PR expression, as well as human epidermal growth factor receptor 2 (HER2) amplification.^31^

Serine metabolism‐associated enzymes, such as phosphoglycerate dehydrogenase (PHGDH), phosphoserine aminotransferase 1 (PSAT1), and 1‐3‐phosphoserine phosphatase (PSPH), are highly expressed in TNBC tumors. Depletion of serine in culture media decreases TNBC cell proliferation. The serine metabolic pathway is essential for the support of TNBC proliferation and metastasis.^32^

Our results indicated that amino acid metabolism plays a role in the phenotypic behavior and unique properties of CSCs.

One of the limitations of this study was the inability of our device to measure proline and cysteine amino acids. Studies have shown that these two amino acids are related to the specific characteristics of cancer stem cells, such as self‐renewal, sphere formation, and resistance.^33^ In addition, mimicking the CSC tumor model by spheroid enrichment approach may only enrich a subset of the CSCs.

The change in the expression of amino acid transporters plays a role in reprogramming cancer cells, which is responsible for the formation of cancer stem cells.^34^ Therefore, for future studies, examining the expression change of these transporters in spheroids and monolayer cells can support the findings obtained from the change of amino acid profiles during the formation of spheroids. In addition, it is necessary to characterize circulating amino acids in patients with breast cancer to indicate whether the in vitro effects of breast CSCs on extracellular amino acids still exist in vivo.

## 5 CONCLUSION

We showed in this study that amino acid levels change during spheroid formation in MCF‐7 and MDA‐MB‐231 cells. These findings suggest a function for the amino acids in the enrichment of spheroid cells with CSC properties in MCF‐7 and MDA‐MB‐231 cell lines.

## AUTHOR CONTRIBUTIONS

**Zahra Ghanbari Movahed:** Conceptualization; Methodology; Writing–original draft. **Maryam M. Matin:** Conceptualization and Supervision; Data analysis; Writing‐review and editing. **Kamran Mansouri:** Conceptualization and Supervision; Data analysis; Writing‐review and editing. **Sajjad Sisakhtnezhad:** Writing–review and editing.

## CONFLICT OF INTEREST STATEMENT

The authors have stated explicitly that there are no conflicts of interest in connection with this article.

## ETHICS STATEMENT

This study was approved by the Research Ethics Committee of the Kermanshah University of Medical Sciences (IR.KUMS.REC.1396.579).

## Data Availability

Data sharing is not applicable to this article as no new data were created or analyzed in this study.
